# Lowered Risk of Nephrotoxicity through Intervention against the Combined Use of Vancomycin and Tazobactam/Piperacillin: A Retrospective Cohort Study

**DOI:** 10.1128/spectrum.00355-21

**Published:** 2021-08-04

**Authors:** Kazutaka Oda, Yumi Hashiguchi, Tomomi Katanoda, Hirotomo Nakata, Hirofumi Jono, Hideyuki Saito

**Affiliations:** a Department of Pharmacy, Kumamoto University Hospital, Kumamoto, Japan; b Department of Infection Control, Kumamoto University Hospital, Kumamoto, Japan; Brigham and Women's Hospital, and Harvard Medical School

**Keywords:** vancomycin, tazobactam/piperacillin, nephrotoxicity, combination

## Abstract

The combined use of vancomycin (VCM) and tazobactam/piperacillin (TAZ/PIPC) is a major risk factor for nephrotoxicity. We sought to evaluate interventions against the combined use of VCM and TAZ/PIPC. This retrospective cohort study involved patients who considered the combined use of VCM and TAZ/PIPC as a treatment. Patients that had either or both antimicrobials replaced were assigned to the intervention group, whereas those who were continued on combination therapy were assigned to the comparison group. The primary endpoint was the incidence of acute kidney injury (AKI). The survival rate of patients on day 30 was evaluated as the secondary endpoint. The comparison and intervention groups were composed of 65 and 68 patients, respectively, and the incidence rates of AKI were 44.6% and 17.6%, respectively. Cox proportional hazard analysis identified the intervention as the only independent factor against AKI development, with a hazard ratio of 0.282 (95% confidence interval [CI], 0.141 to 0.565). For the incidence of AKI of grade greater than 1, the hazard ratio was 0.114 (95% CI, 0.025 to 0.497). The survival rates on day 30 in the comparison and intervention groups were 92.3% and 91.2%, respectively, with a relative risk of 0.988 (95% CI, 0.892 to 1.094). The trough VCM concentration was not associated with the incidence of AKI in patients receiving the combination therapy. This study demonstrated that intervention against the combined use of VCM and TAZ/PIPC can lower the risk of nephrotoxicity.

**IMPORTANCE** The combined use of vancomycin (VCM) and tazobactam/piperacillin (TAZ/PIPC) is a major risk factor for nephrotoxicity. We retrospectively evaluated interventions against the combined use of VCM and TAZ/PIPC. Patients for whom either or both antimicrobials were replaced were assigned to the intervention group (65 patients), whereas those who were continued on combination therapy were assigned to the comparison group (68 patients). The primary endpoint was the incidence of acute kidney injury (AKI). The incidence rates of AKI in the intervention and comparison groups were 44.6% and 17.6%, respectively. Cox proportional hazard analysis identified intervention as the only independent factor against AKI development, with a hazard ratio of 0.282 (95% confidence interval [CI], 0.141 to 0.565). In conclusion, this study demonstrated that intervention against the combined use of VCM and TAZ/PIPC can lower the risk of nephrotoxicity.

## INTRODUCTION

Vancomycin (VCM), a glycopeptide antimicrobial agent, elicits bactericidal activity specifically against Gram-positive bacteria, including resistant strains, such as methicillin-resistant Staphylococcus aureus (MRSA) (1). For decades, numerous studies have been conducted to identify strategies that can minimize nephrotoxicity, and the risk factors for this condition include excessive exposure to VCM, a combination of VCM and a nephrotoxic agent, hypovolemia, and certain comorbidities (2). Tazobactam/piperacillin (TAZ/PIPC) is a beta-lactam antimicrobial agent with minimal risk of nephrotoxicity, at least as a monotherapy; however, the combined use of VCM and TAZ/PIPC was reported as an unexpected nephrotoxic factor in 2014 (3). The underlying mechanism for this has not been elucidated, but a meta-analysis of clinical studies supported this risk (4). Importantly, TAZ/PIPC exhibits broad-spectrum bactericidal activity against Gram-negative bacteria, although it is ineffective against MRSA. This means that TAZ/PIPC can be empirically used in combination with VCM to treat severe infectious diseases before pathogen identification, although its increased nephrotoxicity cannot be ignored. We hypothesized that an intervention to replace VCM with another anti-MRSA agent or TAZ/PIPC with another antimicrobial agent may prevent kidney injury. Studies in children’s hospitals have demonstrated the efficacy of an intervention to avoid the use of concurrent nephrotoxic agents against nephrotoxicity, and it involved the replacement of TAZ/PIPC with cefepime as the first-line drug and strict VCM therapeutic drug monitoring (5, 6). However, while the antimicrobial spectrum pattern of TAZ/PIPC is different from that of cefepime, carbapenems should be discouraged as a fixed alternative because of their extended broad antimicrobial spectrum. Considering the principle of appropriate use of antimicrobial agents, an intervention based on personal direct intervention may be effective in avoiding the combined use of VCM and TAZ/PIPC. Hence, we started the intervention at our hospital in December 2017 to minimize nephrotoxicity for patients being considered to use a combination of VCM and TAZ/PIPC. The aim of this study was to evaluate interventions against the combined use of VCM and TAZ/PIPC.

## RESULTS

**FIG 1:**
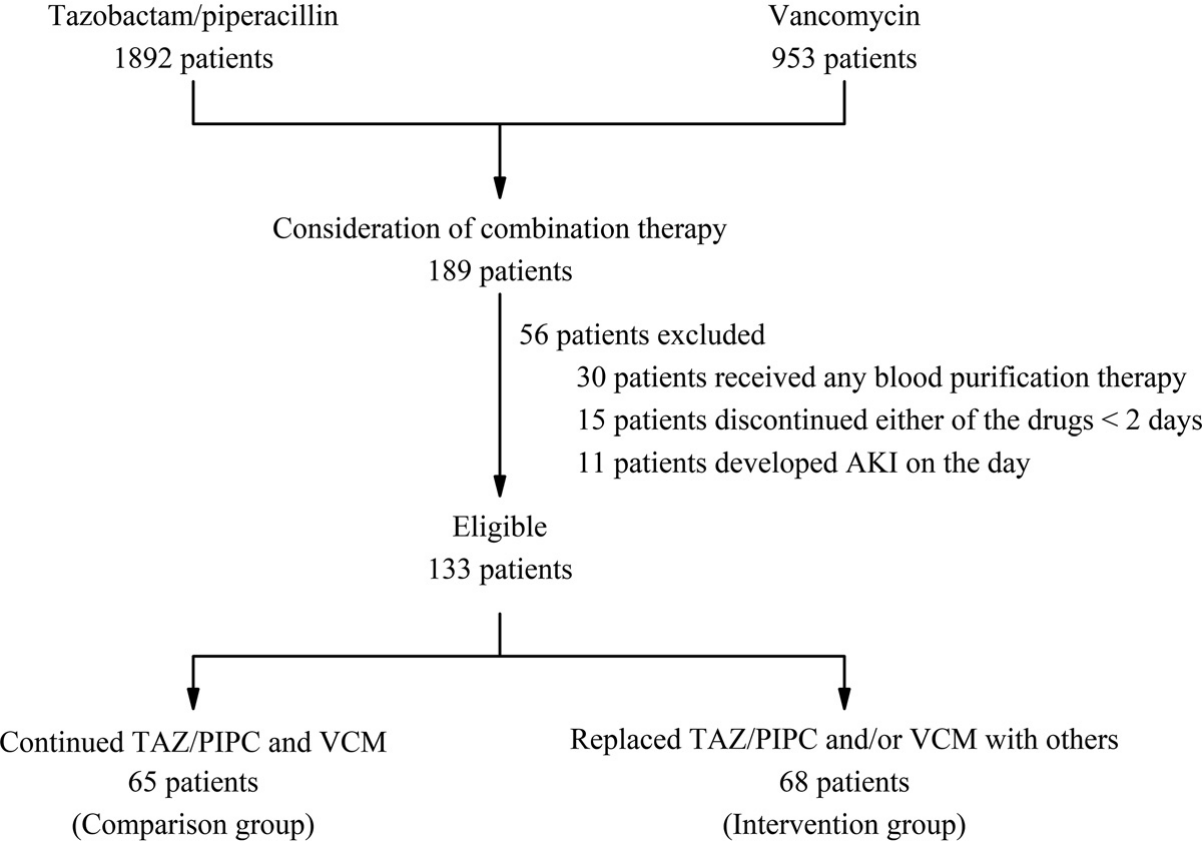
Process for including participants in this study.

Figure 1 shows the process of including the participants. In patients administered TAZ/PIPC (*n* = 1,892) or VCM (*n* = 953), 189 patients considered the combination, 56 patients were excluded based on the exclusion criteria, and the remaining 133 patients were eligible for this study. The comparison and intervention groups were composed of 65 and 68 patients, respectively. In the intervention group, 41/68 (60.3%) of the patients were directly intervened by the pharmacist, whereas the remaining 27 patients replaced either or both antimicrobials at the discretion of the attending physician. Table 1 shows the demographic characteristics of the included patients. VCM was replaced with teicoplanin (a glycopeptide) in 35/68 (51.5%) of the patients, and TAZ/PIPC was replaced with meropenem (a broad-spectrum carbapenem) in 15/68 (22.1%) of the patients or cefepime (a cephalosporin) in 5/68 (7.4%) of the patients. Among those patient cases where meropenem was used as the alternative, 8/15 (53.3%) were further replaced with a narrower-spectrum antimicrobial agent as de-escalation therapy based on susceptibility data for isolated pathogens and other risk factors for antimicrobial resistance. The median duration (range) of the combined use of an anti-MRSA agent (involving VCM) and TAZ/PIPC in the intervention group was 4 (1 to 26) days. In the intervention group, 54/68 (79.4%) of the patients absolutely avoided the combined use of VCM and TAZ/PIPC, while the remaining 14 patients were administered the combination for 4 (1 to 6) days prior to the discontinuation. Patients in the comparison group were administered the combined use of VCM and TAZ/PIPC for 5 (1 to 45) days. In 35.0% (7/20) of patients with pneumonia, MRSA was isolated from the nares or respiratory tract prior to the combination use.

**TABLE 1:** Demographics of included patients

Characteristic	Comparison group (*n* = 65)		Intervention group (*n* = 68)		
	Median	Range	Median	Range	*P* value
Basic property^*a*^					
Male/female (male %)	46/19 (70.8%)		34/34 (50.0%)		0.021
Age, y	66	2–90	67.0	3–83	0.223
Actual body weight, kg	58	31–90	57.0	16–83	0.178
Department of surgery, *n* (%)	37 (56.9%)		29 (42.6%)		0.120
Intensive care unit, *n* (%)	16 (24.6%)		13 (19.1%)		0.530
Diabetes mellitus, *n* (%)	29 (42.6%)		29 (44.6%)		0.862
Hypertension, *n* (%)	25 (36.8%)		28 (43.1%)		0.483
Malignancy, *n* (%)	49 (75.4%)		56 (82.4%)		0.396
Chronic kidney disease, *n* (%)	8 (12.3%)		22 (32.4%)		0.007
Serum albumin, g/dl	2.8	1.5–4.0	3.1	1.5–4.3	0.057
Serum sodium, mmol/liter	138	121–145	139	127–149	0.128
Blood urea nitrogen, mg/dl	15.3	4.3–45.9	15.1	6.2–41.1	0.441
Serum creatinine level, mg/dl	0.69	0.24–2.61	0.75	0.21–2.49	0.223
eGFR, ml/min/1.73 m^2^	86.8	20.6–337.3	74.7	20.6–381.6	0.643
Total bilirubin, mg/dl	0.8	0.2–5.5	0.7	0.2–12.4	0.254
Aspartate transaminase, IU/liter	23	0–255	17	9–293	0.985
Alanine transaminase, IU/liter	22	7–456	21	7–190	0.396
γ-Glutamyl transpeptidase, IU/liter	41	12–1349	49	8–878	0.737
C-reactive protein, mg/dl	7.2	0.0–30.7	5.4	0.0–36.8	0.160
White blood cell, counts × 10^3^/μl	4.0	0.0–31.7	4.7	0.0–32.6	0.550
Red blood cell, counts × 10^6^/μl	3.1	1.6–5.7	3.0	1.8–4.6	0.864
Hemoglobin, g/dl	9.5	5.1–14.4	9.3	4.3–13.6	0.876
Hematocrit, %	28.2	14.9–43.5	28.3	18–40.5	0.769
Platelet, counts × 10^6^/μl	134	4–746	111	5–428	0.490
Max body temperature, °C	38.0	36.4–40.6	38.2	36.4–40.7	0.777
Heart rate, bpm	100	61–163	103	67–174	0.844
Systolic blood pressure, mm Hg	98	59–134	103	60–156	0.082
Diastolic blood pressure, mm Hg	57	20–83	59	40–111	0.124
SOFA score	3	0–8	2	0–6	0.248
Infection					
Chest infection, *n* (%)	12 (18.5%)		12 (17.6%)		1.000
Soft tissue infection, *n* (%)	18 (27.7%)		10 (14.7%)		0.089
Febrile neutropenia, *n* (%)	17 (26.2%)		26 (38.2%)		0.144
Intraabdominal infection, *n* (%)	4 (6.2%)		12 (17.6%)		0.061
Urinary tract infection, *n* (%)	1 (1.5%)		2 (2.9%)		1.000
Others, *n* (%)	9 (13.8%)		4 (5.9%)		0.151
Undiagnosed, *n* (%)	4 (6.2%)		2 (2.9%)		0.444
Bacteremia, *n* (%)	15 (23.1%)		22 (32.3%)		0.252
Exposure					
Nephrotoxin use, *n* (%)	53 (81.5%)		60 (88.2%)		0.336
Loop diuretics, *n* (%)	14 (21.5%)		15 (22.1%)		1.000
NSAIDs, *n* (%)	14 (21.5%)		17 (25.0%)		0.685
Vasopressor, *n* (%)	7 (10.3%)		8 (12.3%)		0.788
Aminoglycosides, *n* (%)	0 (0.0%)		3 (4.4%)		0.245
VCM continued, *n* (%)	65 (100%)		29 (42.6%)		<0.001
First dose, mg/kg	17.2	7.2–41.7	20.8	7.4–33.3	0.151
First 24 h dose, mg/kg	35.2	14.3–64.5	39.7	14.7–61.3	0.968
Maintenance dose, mg/kg/d	30.9	14.3–48.8	30.7	14.7–45.5	0.573
First trough concentration, μg/ml	10.8	4–28.1	10.2	4.9–22.4	0.345
Intervention					
Teicoplanin, *n* (%)	0 (0%)		35 (51.5%)		
Linezolid, *n* (%)	0 (0%)		3 (4.4%)		
Daptomycin, *n* (%)	0 (0%)		1 (1.5%)		
Meropenem, *n* (%)	0 (0%)		15 (22.1%)		
Cefepime, *n* (%)	0 (0%)		5 (7.4%)		
Others, *n* (%)	0 (0%)		9 (13.2%)		
TAZ/PIPC duration, days	10	2–58	7	1–33	0.062
Anti-MRSA agent duration, days	9	2–62	11	10–35	0.107
Combination duration^*b*^, days	5	1–45	4	1–6	<0.001
Outcomes					
Development of AKI, *n* (%)	29 (44.6%)		12 (17.6%)		0.001
AKI grade, *n* (%)					
1	16 (24.6%)		10 (14.7%)		
2	9 (13.8%)		2 (2.9%)		
3	4 (6.2%)		0 (0.0%)		
Survival rate on day 30	60 (92.3%)		62 (91.2%)		1.000

a Bpm, beats per minute; NSAIDS, nonsteroidal anti-inflammatory drugs.

b Duration of the combined use of VCM and TAZ/PIPC.

### Outcomes.

**FIG 2:**
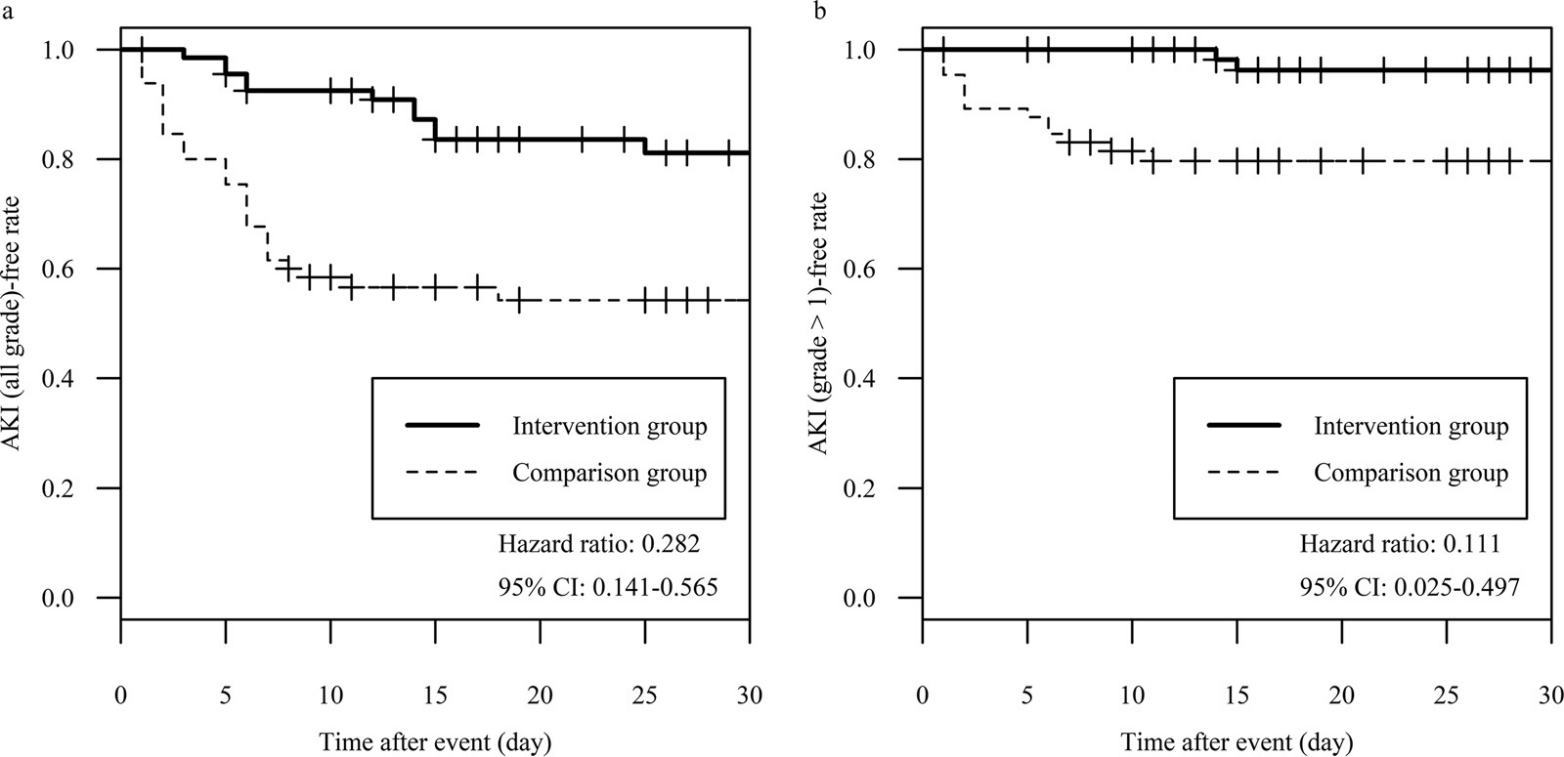
Acute kidney injury (AKI)-free curve for the intervention or comparison groups. The hazard ratios and their corresponding 95% CIs were calculated using Cox proportional hazard analysis. AKI was defined using the KDIGO classification (see Materials and Methods; reference [7]). (a) AKI of all grades. (b) AKI of grade >1.

**FIG 3:**
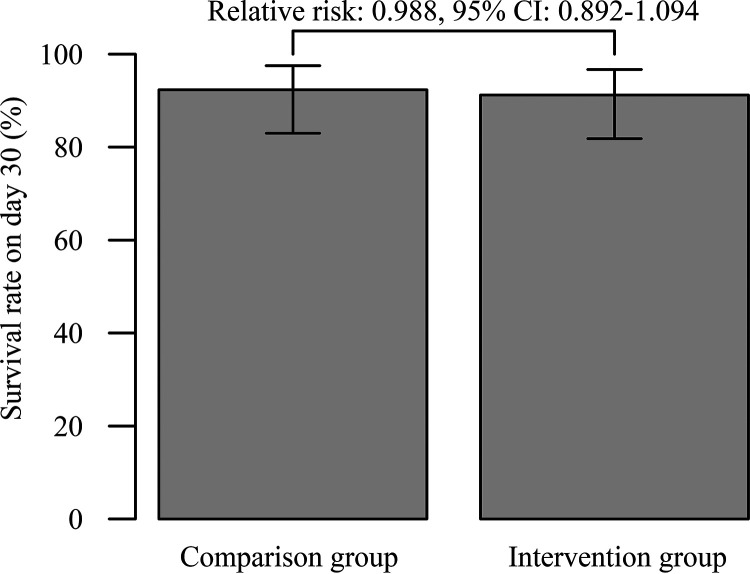
Survival rate on day 30. The lower limit of its 95% CI for the relative risk (vertical lines in the upper part of the bar plots) was outside the noninferiority margin.

Development of acute kidney injury (AKI) of all grades was found in 44.6% (29/65) of the patients in the comparison group and in 17.6% (12/68) in the intervention group. In the univariate analysis, intervention, sequential organ failure assessment (SOFA) score, and individual actual body weight were statistically significant. In the Cox proportional hazard model analysis, the intervention was identified as the only significant factor that lowered the risk of nephrotoxicity, with a hazard ratio of 0.282 (95% confidence interval [CI], 0.141 to 0.565), as shown in the AKI (all grade)-free curve in Fig. 2a. For those with an AKI grade of >1 (comparison group: 20%, 13/65; intervention group: 2.9%, 2/68), intervention, age, and comorbid chronic kidney disease were statistically significant in the univariate analyses. The Cox proportional hazard model analysis revealed that the intervention was a significant factor in lowering the nephrotoxicity risk, with a hazard ratio of 0.111 (95% CI, 0.025 to 0.497), as displayed in the AKI (grade > 1)-free curve in Fig. 2b. Aging was also a significant factor, with a hazard ratio of 0.973 (95% CI, 0.952 to 0.995). Figure 3 shows the survival rate of patients on day 30, and the values in the comparison and intervention groups were 92.3% and 91.2%, respectively (the Kaplan-Meier curve is shown in Fig. S1 in the supplemental material). The relative risk was 0.988 (95% CI, 0.892 to 1.094), where the lower limit of its 95% CI was outside the noninferiority margin. The composite endpoint is shown in Fig. S2. In the subgroup analysis (data not shown) of the intervention group with the replacement of VCM with teicoplanin against the comparison group, the hazard ratio for AKI of any grade was 0.460 (95% CI, 0.217 to 0.972), and the relative risk for the survival rate on day 30 was 0.960 (95% CI, 0.836 to 1.102).

**FIG 4:**
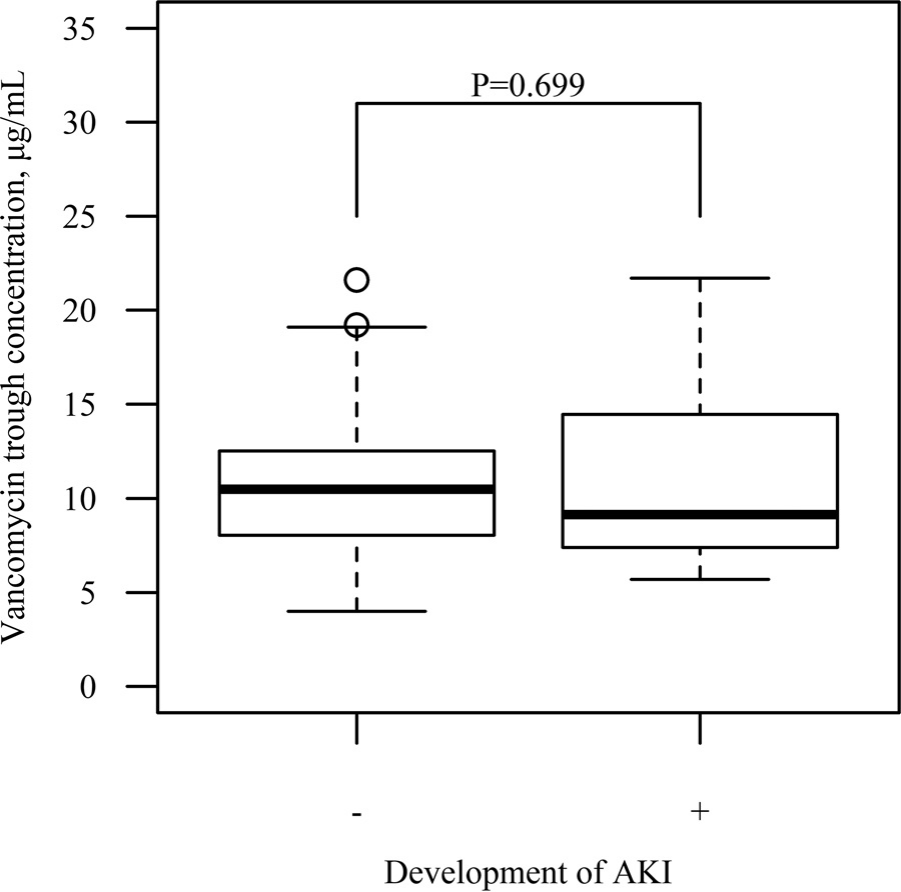
Subgroup analysis of the comparison group for vancomycin trough concentration and AKI development. Vancomycin trough concentration was measured in 47 patients; among them, 16 patients developed AKI (right column). The number of maintenance dosing interval (6 h, 8 h, 12 h, and 24 h) in the group without/with AKI was 1/0, 9/5, 19/10, and 2/1, respectively.

Figure 4 shows an analysis of the possible attribution of vancomycin trough concentration to the development of AKI in the comparison group. The median concentration in the subgroup of patients with AKI (*n* = 16, 9.2 μg/ml) was not significantly different from that in the subgroup of patients without AKI (*n* = 31, 10.5 μg/ml, *P = *0.699). The *P* value for diabetes mellitus and estimated glomerular filtration rate (eGFR) was <0.05 in the univariate analysis, but they were not statistically significant in the multivariate logistic regression analysis.

## DISCUSSION

This study showed that an intervention against the combined use of VCM and TAZ/PIPC lowered the risk of nephrotoxicity (hazard ratio = 0.282). The demographics of the patients were comparable between the groups, but a few variables that showed differences should be discussed. The intervention group showed a significantly higher ratio of female patients, chronic kidney disease as a comorbidity, and anti-MRSA agent duration. These are known to be risk factors for nephrotoxicity (1, 7, 8). Serum albumin, blood pressure, and its attributed SOFA score are likely biased, but not significant. These potential biases might have affected the results because these factors were considered risk factors for nephrotoxicity (9, 10), although the differences in the median values between the groups were likely to be small. Collectively, the included patients and their subsequent assignment could be adequate to evaluate the purpose of this study.

The possibility of elevated creatinine levels due to the inhibition of tubular excretion by TAZ/PIPC has been reported, where the necessity to reconsider the definition of AKI was addressed as a concern (11, 12). An elevation in the serum creatinine level by approximately 0.2 to 0.3 mg/dl after TAZ/PIPC monotherapy has been reported (13, 14). Thus, the patients in this study would meet the definition of AKI with kidney disease improving global outcomes (KDIGO) grade 1. However, the intervention group showed a significant association with AKI development even in patients with a KDIGO grade of ≥2 (≥2-fold elevated serum creatinine level, hazard ratio = 0.111; Fig. 2b). This result was unlikely to be consistent with its ability to inhibit the tubular excretion of creatinine. As for the secondary endpoint, we have provided the survival rate on day 30 (Fig. 3), although noninferiority was not demonstrated. One male patient in the intervention group died due to an acute exacerbation of pneumonia within 24 h after the replacement of TAZ/PIPC with meropenem, so it was difficult to assess the patient for intervention-related survival. He had a vasopressor, comorbid carcinoma, an eGFR of 77.3 ml/min/1.73 m^2^, and a SOFA score of 5. If the patient could be excluded from the evaluation for the secondary endpoint, the relative risk for the survival rate on day 30 was 1.002 (95% CI, 0.909 to 1.105), which could mean noninferiority. Hence, interventions against the combined use of VCM and TAZ/PIPC can be promoted for safe medical care, and we can recommend our interventions (see Materials and Methods).

Teicoplanin is a glycopeptide antimicrobial agent that can elicit the same antibiogram pattern as VCM but has less nephrotoxicity (15). The intervention group in this study showed significantly less nephrotoxicity, which was also observed in the intervention group that only involved patients who replaced VCM with teicoplanin (hazard ratio = 0.460). Recent studies have demonstrated an allowable risk of nephrotoxicity with the teicoplanin and TAZ/PIPC combination but have not reported any outcomes regarding treatment (16, 17). Moreover, we have provided the survival rate on day 30 in the entire intervention group, while that in the limited intervention group using teicoplanin (35 patients) was 0.960 (95% CI, 0.836 to 1.102). Although the lower limit of its 95% CI was outside the noninferiority margin defined in this study, we recruited diverse participants without limitations on the nature of their infections or the severity of their illness. Considering the comparable treatment success rate among patients treated with VCM and teicoplanin in a previous meta-analysis (15), teicoplanin can be an alternative to VCM in various situations when considering the combination of TAZ/PIPC.

Meropenem and cefepime have been assessed as alternatives to TAZ/PIPC (18, 19), but the possible elevated risk of nephrotoxicity has been reported in a meta-analysis where a large heterogeneity among studies was observed (4). This elevated risk of nephrotoxicity by meropenem may imply the presence of a critical illness. Several studies in patients receiving intensive care have reported no elevation in the risk of nephrotoxicity (20–26). To interpret this discrepancy, the mechanism of nephrotoxicity of the combined use of VCM and TAZ/PIPC should be determined, as there are various risks of AKI in patients under intensive care. In contrast, a report on the protective effect of TAZ/PIPC against nephrotoxicity in an experimental mouse model should be considered (27). Taken together, this suggests that meropenem or cefepime can be an alternative to TAZ/PIPC, especially for patients in intensive care units who require maximal attention.

This study indicated no association between VCM trough concentration and AKI development (Fig. 4) in patients receiving the combination therapy; however, this should be further evaluated. Comparable trough concentrations between VCM cohorts with and without TAZ/PIPC have been reported, although multivariate analyses demonstrated that vancomycin trough concentration is a statistically significant risk factor for AKI independent of the concomitant use of TAZ/PIPC in the VCM cohort (28–34). A previous study indicated that VCM trough concentration is a negative risk factor for nephrotoxicity in a small cohort (*n* = 15) (35). Considering the pharmacologic principle, vancomycin trough concentration as an exposure surrogate may be attributed to AKI development in a concentration-dependent manner, indicating that the threshold can be lower than the therapeutic target. Because the surrogate trough concentration for therapeutic area under the concentration-time curve (AUC) (>400 μg · h/ml) is approximately 10 μg/ml, a trough concentration lower than that observed in the study (mean = 9.2 μg/ml in the group that developed AKI) should be a considered a subtherapeutic AUC. A previous study supported this hypothesis, that is, AUC-guided dosing of vancomycin did not contribute to the reduction in AKI risk (36).

This study had some limitations. First, we analyzed different kinds of variates between the cohorts, which were unmatched in the propensity scores. When we attempted to match the propensity scores, there were only 32 participants (16 each); thus, the cohorts did not reach the sample size and lacked detectable power. Second, we pooled different kinds of interventions as one cohort. Therefore, the hazard ratio specific to each intervention should be further analyzed. Third, direct intervention by a pharmacist was not developed as part of the protocol. Hence, standardized interventions should be developed.

## CONCLUSION

This study indicated that the intervention against the combined use of VCM and TAZ/PIPC can lower the risk of nephrotoxicity.

## MATERIALS AND METHODS

### Ethics, study design, and participants.

This study was conducted in accordance with the Declaration of Helsinki and national and institutional standards after approval from the Institutional Review Board of Kumamoto University Hospital (approval no. 2413). The interventions against the combined use of VCM and TAZ/PIPC were started in December 2017 to avoid nephrotoxicity, and then we retrospectively evaluated the interventions as a cohort study after August 2020. Informed consent was obtained from each participant in an opt-out manner through a description of our study on our website with the opportunity to drop out from this study. The study period was from December 2017 to July 2020. The following inclusion criteria were used: (i) patients who received VCM and required additional TAZ/PIPC or other broad-spectrum agents, (ii) patients who received TAZ/PIPC and required additional VCM or another anti-MRSA agent, and (iii) patients who were prescribed the combined use of VCM and TAZ/PIPC as an empirical therapy. The following exclusion criteria were used: (i) patients undergoing any blood purification therapy (e.g., continuous/intermittent hemodialysis and/or filtration, plasma exchange) during the combination therapy, (ii) patients who developed AKI on the day of possible inclusion, or (iii) use of a combination of any broad-spectrum agent and an anti-MRSA agent for less than 2 days before meeting the primary or secondary endpoint.

### Intervention.

The use of TAZ/PIPC, carbapenems, and any anti-MRSA agents including VCM has been restricted and monitored as a part of antimicrobial stewardship prior to the interventions. A trained pharmacist (the first author), who was certified as an infectious disease chemotherapy pharmacist by the Japanese Society of Chemotherapy and certified as an infection control pharmacist specialist by the Japanese Society of Hospital Pharmacists dispensed the interventions. The pharmacist optimized the antimicrobial therapy in the hospital during half of the work time and offered pharmacy services in the intensive care unit in the remaining half of the work time until March 2019. The pharmacist was then shifted to the antimicrobial stewardship team full time during weekdays, except at night and on weekends, from April 2019. The intervention was administered based on direct personal interventions for patients who were considering the combination. For example, direct intervention included reviewing the antimicrobial regimen, presence of possible pathogens, and concomitant nephrotoxic factors. In cases in which an antimicrobial of broad-spectrum equivalent to TAZ/PIPC (for Gram-negative bacteria and anaerobes) was needed, carbapenems or other anti-MRSA agents were suggested as suitable alternatives, while other cases suggested cephalosporin and/or metronidazole as pivotal alternatives. If MRSA was isolated from the nares or respiratory tract of patients with pneumonia prior to the combination use, daptomycin was avoided as the alternative. The antibiotic regimens were further tailored according to pathogens isolated. In cases when the combination had already been run at the off-duty time of the pharmacist, direct intervention was occasionally attempted on the next duty time. As an indirect intervention, the pharmacist provided a handy manual for the appropriate use of antimicrobial agents, which was accompanied by information on the increased nephrotoxicity risk of the combination, to physicians, pharmacists, and relevant clinical workers. Patients who were continued on the combined use of VCM and TAZ/PIPC initiated on off-duty time or despite the interventions (including failing to convince the prescriber or prescription occurred outside duty time) were assigned to the comparison group whereas those for whom either or both VCM and/or TAZ/PIPC were replaced after the pharmacist’s direct intervention or the attending physician’s discretion were assigned to the intervention group.

### Outcomes.

The incidence rate of AKI was evaluated as the primary endpoint, where the KDIGO classification was used for the definition of AKI (7). The follow-up period was from the day of drug administration up to 14 days after the end of drug administration. This was analyzed using the Cox proportional hazard model, including possible nephrotoxic factors determined via univariate analyses between subgroups with and without AKI. A *P* value of <0.05 was used as the statistical significance cutoff in the unpaired *t* test or Wilcoxon signed-rank test for continuous variables or Fisher’s exact test for categorical variables. The survival rate on day 30 was evaluated as the secondary endpoint, where the relative risk was used. Because the relative risk evaluation required noninferiority, we set the noninferior margin to 0.1, where the lower limit of the 95% CI had to be greater than 0.9. The composite endpoint with AKI or survival rate on day 30 was additionally evaluated in a time-to-event manner. The possible attribution of VCM trough concentration to nephrotoxicity in the combined use of VCM and TAZ/PIPC was also evaluated. The subgroup for this evaluation was extracted from the patients in the comparison group with measured VCM trough concentrations, and patients who developed AKI before or on the day of measurement were removed from the subgroup.

### Sample size.

Based on the findings of a previous study, that is, an AKI risk of 25.8% and 6.7% with the combination and comparator, respectively (37), with an assumed detection power of 0.8, the sample size was calculated as 63 participants in each group for the primary endpoint.

### Statistical calculation.

R version 3.6.3 (https://www.r-project.org/) was used to perform all statistical analyses, where the “survival” and “party” packages were used for the Cox proportional hazard analysis and the survival classification and regression tree analysis, respectively.

### Data availability.

The data set used for this study is provided in Data Set S1 in the supplementary material.
