# Supplementary material for: Lowered Risk of Nephrotoxicity through Intervention against the Combined Use of Vancomycin and Tazobactam/Piperacillin: A Retrospective Cohort Study
Source: Microbiol Spectr. 2021 Aug 4;9(1):10.1128/spectrum.00355-21. doi: 10.1128/spectrum.00355-21 (PMC8552786; doi:10.1128/spectrum.00355-21)
Supplement: SUPPLEMENTAL FILE 1 — Supplemental material. Download SPECTRUM00355-21_Supp_1_seq8.pdf, PDF file, 0.2 MB [file spectrum00355-21_supp_1_seq8.pdf]

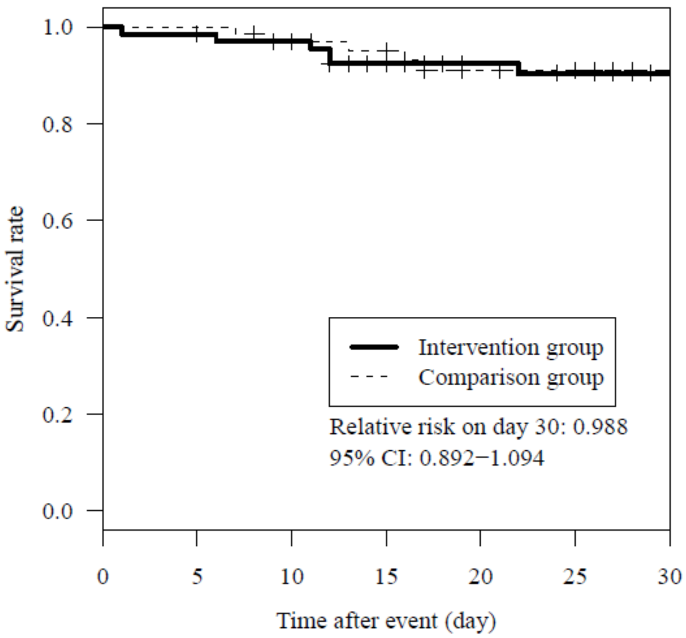

Fig. S1 Kaplan-Meier curve of survival rate for the intervention or comparison groups corresponding to Figure 3.

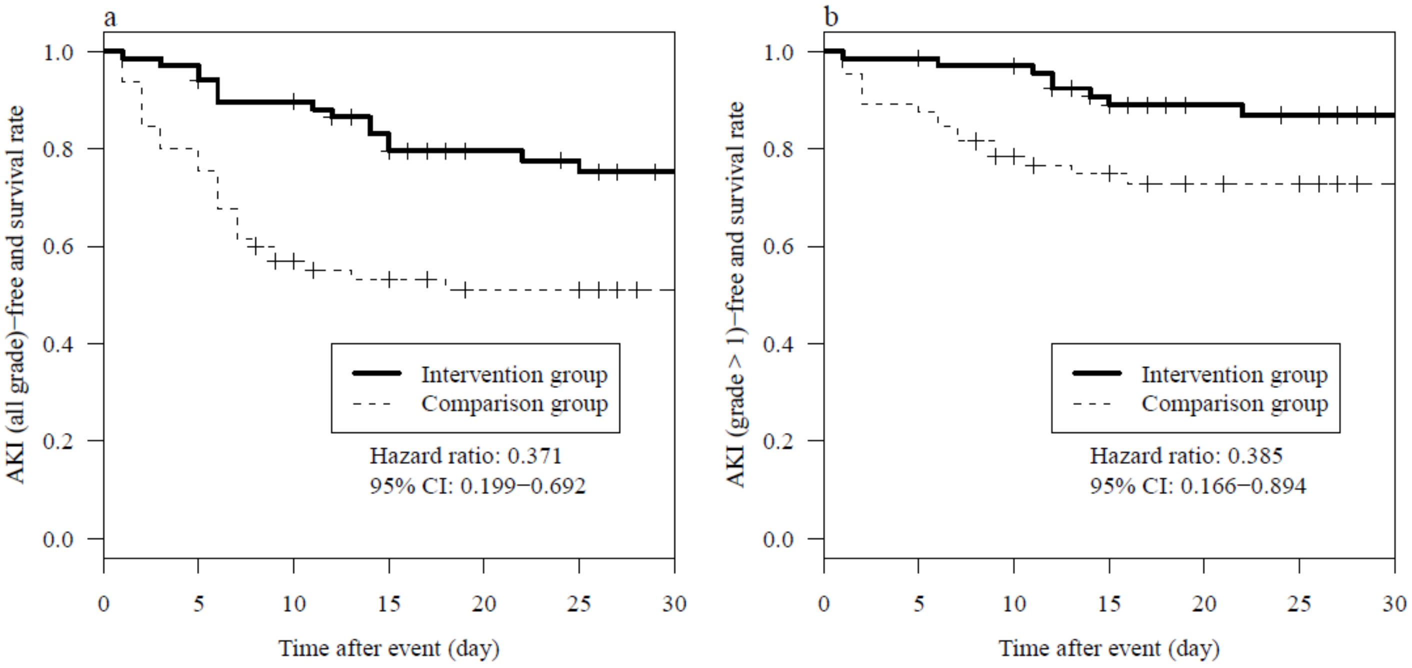

Fig. S2 Composite end point (acute kidney injury (AKI)-free and survival) curve for the intervention or comparison groups. The hazard ratios and their corresponding 95% CIs were calculated using Cox-proportional hazard analysis. AKI was defined using the KDIGO classification. [1]

a: AKI of all grades. b: AKI of grade > 1.

Data S1 *Intervention*, intervention performed = 1; *Change*, replaced either or both of tazobactam/piperacillin and/or vancomycin with others = 1; *AKIAllGrade*, acute kidney injury of any grade occurred = 1; *Surgery*, department of surgery = 1; *ICU*, admitted into intensive care unit = 1; *VCM*, vancomycin used = 1; *TEIC*, teicoplanin used = 1; *LZD*, linezolid used = 1; *DAP*, daptomycin used = 1; *FirstDosemg*, first dose (mg); *FirstDosemgkg*, first dose per body weight of one kilogram (mg/kg); *Dose24mg*, cumulative dose during first 24 hours (mg); *Dose24mgkg*, cumulative dose during first 24 hours per body weight of one kilogram (mg/kg); *MDmg*, maintenance dose per day (mg); *MDmgkg*, maintenance dose per body weight of one kilogram per day (mg/kg/day); *INTERVAL*, dosing interval; *CONC*, measured vancomycin concentration at trough; *MeasurementDay*, days until measurement of vancomycin concentration; *Combidays*, days of the combined use of tazobactam/piperacillin and vancomycin or any anti-MRSA agents; *TAZPIPCdays*, days of using tazobactam/piperacillin; *MRSAagentsdays*, days of using any anti-MRSA agents; *Nephrotoxicin*, any nephrotoxic agent used = 1; *Vaso*, vasopressor used = 1; *DM*, comorbid diabetes mellitus = 1; *HT*, comorbid hypertension = 1; *Carcinoma*, comorbid carcinoma = 1; *Loopinfusion*, intravenous loop diuretics used = 1; *Loopall*, intravenous or oral loop diuretics used = 1; *Diuretics*, any diuretics used = 1; *NSAIDs*, non-steroidal anti-inflammatory drugs used = 1; *Ags*, any aminoglycosides used = 1; *ALB*, serum albumin level (g/dL); *Na*, serum sodium level (mmol/L); *K*, serum potassium level (mmol/L); *BUN*, blood urea nitrogen level (mg/dL); *Scr*, serum creatinine level (mg/dL); *eGFR*, estimated glomerular filtration rate (mL/min/1.73 m^2); *CKD*, comorbid chronic kidney injury = 1; *Tbil*, total bilirubin level (mg/dL); *ASP*, aspartate transaminase (IU/L); *ALT*, alanine transaminase (IU/L); *LD*, lactate dehydrogenase (IU/L); *γGTP*, gamma glutamyl transpeptidase (IU/L); *CRP*, C-reactive protein (mg/dL); *WBC*, white blood cells (counts $\times 10^3$/μL); *RBC*, red blood cells (counts $\times 10^6$/μL); *Hgb*, hemoglobin (g/dL); *Hct*, hematocrit (%); *PLT*, platelet (counts $\times 10^6$/μL); *Survival*, survival days (if not confirmed = 1095); *Survival60*, survival on day 60 = 1; *Survival30*, survival on day 30 = 1; *Discharge*, discharged from hospital = 1; *Deathinhospital*, died before discharge = 1; *AKIGrading*, grade of acute kidney injury with KDIGO classification[1]; *AKIGrade234*, acute kidney injury with KDIGO classification[1] > 1 = 1; *AKIfreeSurvival*, survival days without acute kidney injury confirmed (if not confirmed = 1095); *AKIfreeSurvivalCensAllGrade*, the values of 1095 for *AKIGradefreeSurvival* are replaced with censored day; *AKIfreeSurvivalCensGrade234*, the values of 1095 for survival days without acute kidney injury with KDIGO classification[1] > 1 (if not confirmed = 1095) are replaced with censored day; *CompositeAll*, both acute kidney injury of all grade and 30-days survival = 1; *CompositeGrade234*, both acute kidney injury with KDIGO classification[1] > 1 and 30-days survival = 1; *CompositeCensAllGrade*, days for composite end point including acute kidney injury of all grade and censored day; *CompositeCensGrade234*, days for composite end point including acute kidney injury with KIDGO classification[1] > 1 and censored day; *AKIonVCMconcEval*, vancomycin concentration measured and developed acute kidney injury of all grade = 1; *Tmax*, max body temperature (°C); *HR*, heart rate (bpm); *SBP*, systolic blood pressure (mmHg); *DBP*, diastolic blood pressure (mmHg); *SOFA*, sequential organ failure assessment score; *MAP*, mean arterial pressure (mmHg); *SEX*, female = 1; *Age*, years after birth; *Height*, body height (cm); *BW*, body weight (kg); *BloodCulturePositive*, bacteremia = 1; *FN*, febrile neutropenia = 1; *INTRAABDO*, intraabdominal infection = 1; *UNK*, unknown infection = 1.

References

1. KDIGO clinical practice guideline for acute kidney injury. Kidney Int. 2012;2:1–138. https://doi.org/10.1038/kisup.2012.7
